# A new tool called DISSECT for analysing large genomic data sets using a Big Data approach

**DOI:** 10.1038/ncomms10162

**Published:** 2015-12-11

**Authors:** Oriol Canela-Xandri, Andy Law, Alan Gray, John A. Woolliams, Albert Tenesa

**Affiliations:** 1 The Roslin Institute and Royal (Dick) School of Veterinary Studies, The University of Edinburgh, Easter Bush Campus, Edinburgh EH25 9RG, UK; 2 EPCC, The University of Edinburgh, Edinburgh EH9 3FD, UK; 3 MRC HGU at the MRC IGMM, University of Edinburgh, Edinburgh EH4 2XU, UK

## Abstract

Large-scale genetic and genomic data are increasingly available and the major bottleneck in their analysis is a lack of sufficiently scalable computational tools. To address this problem in the context of complex traits analysis, we present DISSECT. DISSECT is a new and freely available software that is able to exploit the distributed-memory parallel computational architectures of compute clusters, to perform a wide range of genomic and epidemiologic analyses, which currently can only be carried out on reduced sample sizes or under restricted conditions. We demonstrate the usefulness of our new tool by addressing the challenge of predicting phenotypes from genotype data in human populations using mixed-linear model analysis. We analyse simulated traits from 470,000 individuals genotyped for 590,004 SNPs in ∼4 h using the combined computational power of 8,400 processor cores. We find that prediction accuracies in excess of 80% of the theoretical maximum could be achieved with large sample sizes.

## Abstract (web summary)

Availability of computing power can limit computational analysis of large genetic and genomic datasets. Here, Canela-Xandri, *et al*. describe a software called DISSECT that is capable of analyzing large-scale genetic data by distributing the work across thousands of networked computers.

## Graphical abstract

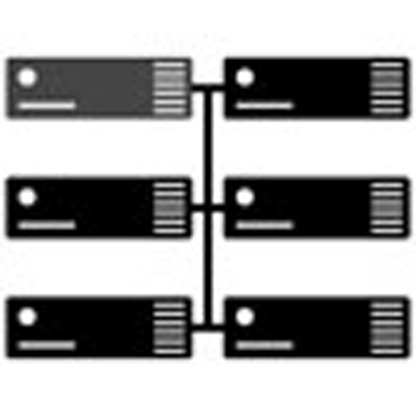

The astonishing rate at which genomic and genetic data are generated is rapidly propelling genomics and genetics research into the realm of ‘Big Data'^1^. This great opportunity is also becoming a big challenge, because success in extracting the useful information contained within these data will depend on our ability to analyse extremely large data sets with the most powerful statistical methods. The computational problems associated with ‘Big Data' become critical when, for instance, fitting mixed-linear models (MLMs) and performing principal component analyses (PCA)^2^,^3^,^4^,^5^,^6^,^7^,^8^,^9^. These analyses are used in a wide range of fields ranging from predictive medicine and epidemiology, to animal and plant breeding, and pharmacogenomics. However, these calculations are so computationally expensive that, when applied to large data sets, one typically resorts to approximations^3^,^8^, restricts the applicability to particular cases (for example, when the number of markers is small compared with the available sample size^9^) or need at least one highly computationally demanding step such as performing the eigen decomposition of the relationship matrix^5^. These ‘workarounds' are non-scalable and therefore could be impractical with increasingly large data sets.

As has been effectively demonstrated in other fields^1^, such limitations can be overcome through moving to software capable of combining the computational power of thousands of processor cores distributed across the nodes of compute clusters and large supercomputers. To address this need, we developed DISSECT (http://www.dissect.ed.ac.uk), a new, highly scalable and freely available tool that is able to perform a large variety of genomic analyses across huge numbers of individuals. For increased versatility, the software also runs on single compute nodes (for example, desktops, regular workstations or single compute nodes on a cluster). Here we describe the methods underpinning our tool and demonstrate its usefulness by addressing the challenge of predicting phenotypes from genotype data in unrelated humans.

Phenotypic prediction is of central interest to disciplines such as quantitative genetics, animal breeding or human medicine and is one of the driving forces behind large-scale genotyping and sequencing projects in a wide range of species^10^,^11^,^12^,^13^,^14^. Despite considerable efforts, predicting complex traits in unrelated humans has been an elusive goal^12^,^15^. Accurate prediction of complex traits is expected to be strongly dependent on the availability of sufficiently large data sets^11^,^15^,^16^ and the capacity to analyse all these data together, which makes this an ideal challenge to showcase DISSECT's capabilities. We therefore simulate a cohort of half a million individuals and use DISSECT and the aggregated power of 8,400 processor cores to analyse it. We show that MLMs could be used to predict quantitative traits with increasing accuracy as the sample size of the training cohort increases and achieve over 80% of the theoretical maximum accuracy when the training cohort has 470,000 individuals. We study different scenarios where the genotyping array contains only ∼20% of the quantitative trait nucleotides (QTNs) together with non-casual single-nucleotide polymorphisms (SNPs), contains all the QTNs together with non-casual SNPs or contains only the QTNs. The improvement in prediction accuracy obtained by including all the QTNs in the array is smaller than by removing the non-causal SNPs from the array, thus indicating the strong detrimental effect that the noise introduced by the non-causal SNPs has on prediction accuracy, even when using large sample sizes.

## Results

### Overview of DISSECT

**Figure 1:**
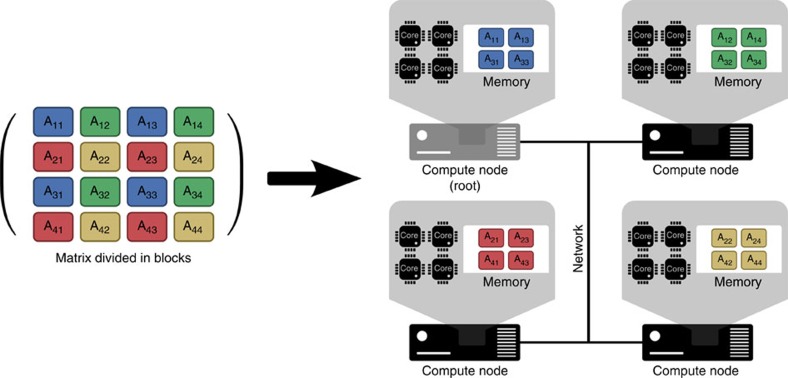
Data distribution among compute nodes. Single compute nodes have a small number of compute cores and a limited amount of memory. This introduces a limit on the dimensions of the matrices that can be analysed by a single compute node, which in turn affects the sample sizes that can be used in common genomic analysis. To overcome the memory and computational capacity limitations, DISSECT decomposes the matrices into blocks and distributes them between networked compute nodes following a two-dimensional cyclic distribution. Each node performs computations on local data and shares data with other nodes through the network connection when the algorithm requires it. The root node coordinates the other nodes, and collects and distributes inputs and outputs when required. This approach allows great scalability, as it is not restricted by the computational limits of a single compute node.

Commonly used statistical analyses of genetic and genomic data are computationally intensive due to the requirement to perform different types of matrix operations. The computational requirements (that is, the compute and memory capacity that is required to perform linear algebra operations on these matrices) are usually a super-linear function of the number of markers and samples available. Therefore, the computational needs for the analysis of increasingly large data sets can rapidly surpass the computational capacity of single compute nodes (Fig. 1). DISSECT is designed to overcome these compute and memory limitations by taking advantage of the aggregate power of the thousands of processor cores and memory that are distributed across clusters of networked compute nodes. For this purpose, it distributes the available data over the multiple nodes using a two-dimensional block-cyclic distribution scheme^17^ (Fig. 1 and Supplementary Note 1). This is convenient, because it achieves a good load balance by splitting the work reasonably evenly among the nodes available, maximizing efficiency and minimizing run time. At any given time, each node has access to only a small portion of the data on which it performs local computations. When the algorithm requires access to blocks of data currently held on other nodes, the nodes communicate to coordinate data redistribution (Supplementary Note 1). In addition, one node (the ‘root' node) takes on the role of coordinating the work of all the other nodes and of performing small summarizing computations. If data sets are small enough to fit in the available memory of the root node, then the root node also handles the data input and output. However, when this is not possible, because the volume of data exceeds the available memory of the root node, then the processes of data loading or storing are also distributed across multiple nodes (Supplementary Note 1).

As the cores on a node cannot directly access data on the other nodes, the computational approach of distributing the data between nodes is necessarily more involved than parallelization of software that uses multiple cores within a single node. In addition, the distribution of workload introduces a relative loss of computational efficiency and scalability, because nodes need to communicate, with overheads determined by the speed of the network connection. Because of this, increasing the number of nodes does not guarantee a proportional reduction of computational time (Supplementary Fig. 1 shows the scalability of DISSECT as a function of the number of processor cores used). However, the broad applicability of this approach enables the analysis of data sets of sizes for which analysis is infeasible using the limited memory and computing capacity of a single compute node. Importantly, no mathematical approximation is required.

DISSECT was written in the programming language C++, using routines from the MPI and BLACS libraries, to handle the data distribution and the communication between nodes. The basic linear algebra computations are based on the ScaLAPACK^17^ libraries, which ensures optimal computational performance when using a performance-optimized implementation such as the Intel Math Kernel Library. DISSECT can be used on computer clusters the size of which may vary from a few tens or hundreds of processor cores to large supercomputers with hundreds of thousands of cores. The sole requirement is that an MPI implementation be available on the machine. Our software also allows the user to take full advantage of multi-core capabilities on more modest, single-node workstations with a performance similar to software designed for running on single compute nodes (Supplementary Fig. 2). DISSECT is as easy to use as other commonly used software such as GCTA^18^ or PLINK^19^ (see Supplementary Note 1) even when running on large supercomputers.

DISSECT implements several highly computational demanding analyses. Some of the most relevant are as follows: computing genetic relationship matrices; performing PCA for studying population structure in large data sets; fitting univariate MLMs; fitting bivariate MLMs, which greatly increase power to detect pleiotropic loci^20^, but require a computational time that is rougly eight times bigger than fitting univariate MLMs to data sets of the same size; regional MLM fitting for studying the accumulated variance explained by the alleles within genomic regions^21^,^22^, each region having similar computational cost regardless of the number of SNPs fitted but requiring an independent fit; and standard regression models with very large number of fixed effects (for example, fitting the markers of a whole chromosome as fixed effects when extremely large sample sizes are available). DISSECT also allows other computationally less demanding analyses such as the prediction of individual phenotypes from estimated marker effects (that is, polygenic scores^23^) or standard genome-wide association study (GWAS) analyses. Furthermore, it also implements optimized routines similar to those found in GEMMA^5^ based on performing the eigen decomposition of the genetic relationship matrix for MLM analysis. These routines allow DISSECT to run analyses much faster when the user wishes to fit several MLM in the same population (see the Supplementary Note 1 for a more detailed description of the analyses).

### Computational performance

**Figure 2:**
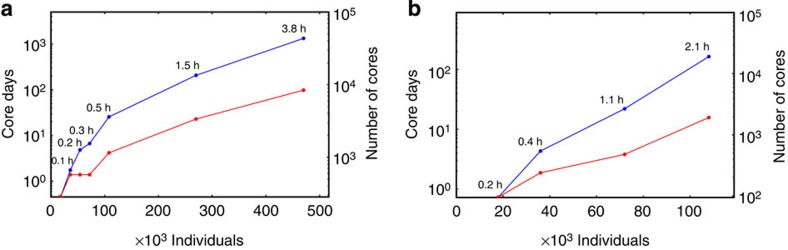
Computational requirements for MLM and PCA. Computational time (blue lines, left axis) and number of processor cores used (red lines, right axis) in log scale for (**a**) MLM and (**b**) PCA analyses as a function of sample size. Core days is the amount of time in days required to complete the analyses multiplied by the number of cores used. It is a rough estimate of the computational time a single computer with a single core would require to perform the analyses if DISSECT scaled perfectly (that is, there was no computational performance penalization due to communication between computer nodes). Labels over the blue dots indicate the real time used for each analysis.

We performed MLM and PCA analyses using simulated cohorts (Supplementary Methods) of different sample size (*N*; Fig. 2), to demonstrate the computational capabilities of DISSECT. We selected these two examples, because they are very computationally demanding analyses, requiring a running time of O(N^3^). The analyses were run on the UK National Supercomputing Service (ARCHER), a supercomputer with 4,920 computer nodes containing 9,840 processors with 12 cores each (that is, a total of 118,080 cores available). DISSECT was able to fit, after eight iterations, an MLM to a sample of 470,000 individuals and 590,004 SNPs in less than 4 h using the aggregated power of 8,400 cores and a total of ∼16 TB of memory (∼2 GB of memory per core; Supplementary Fig. 3). The running time included estimation of the variances using REML^24^,^25^, best linear predictions of the individual's genetic values and best linear predictions of SNP effects^18^,^26^. If we disregard the computational overhead of communication between nodes, we can roughly estimate the computational time required by a computer with one core, to complete the analysis by multiplying the number of used cores with the computation time (core hours). In this situation, the MLM fit would need 3.6 years (Fig. 2a). Performing a PCA for 108,000 individuals and 590,004 SNPs required ∼2 h using 1,920 cores. That is, arround ∼4,000 core hours, which would be equivalent to ∼160 days of computation on a single core (Fig. 2b). All these results show both the high computational demands required for performing these analyses and the ability of DISSECT to perform them.

### Prediction results with huge sample sizes

**Figure 3:**
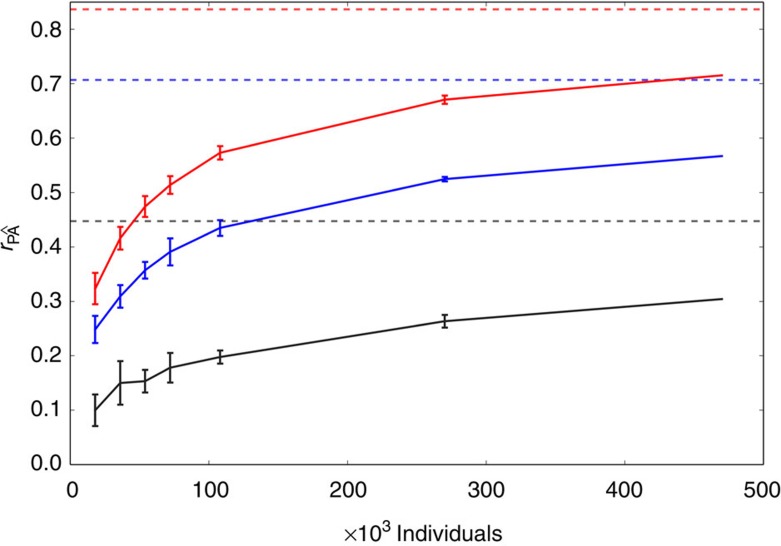
Prediction accuracy of MLM as a function of sample size and heritability. Correlation between true (P) and predicted phenotypes () as a function of cohort size for a trait determined by 10,000 QTNs. Black, blue and red curves represent heritabilities of 0.2, 0.5 and 0.7, respectively. Constant dashed lines indicate the theoretical maximum achievable for each heritability. Error bars are two times the s.d. over 6 replicas (470,000 individuals case has only 1 replica).

We tested the accuracy of phenotypic prediction from genotype data when large numbers of individuals are available. To this end, more than half a million SNP genotypes for half a million individuals were simulated based on linkage disequilibrium patterns and allele frequencies from the Hapmap CEU population. Then, we simulated several quantitative traits by using both different heritabilities (*h*^2^) and numbers of QTNs. We first assumed a situation where only ∼20% of the QTNs were in the genotyping array. In each case, we divided the cohort into two subsets: one for training the models and another for validating the predictions (Supplementary Methods). Predictions were based on the effects of all available SNPs estimated jointly from the MLM fit. As expected, prediction accuracy increased with the heritability of the trait and the size of the training data set (Fig. 3). The MLM efficiently captured the effects of large numbers of genotyped and ungenotyped QTNs. Simulated traits determined by 10,000 QTNs (Fig. 3) gave very similar results to traits determined by 1,000 QTNs (Supplementary Fig. 4). Importantly, high accuracies were only achieved when large numbers of individuals were used to train the prediction model. For instance, training the MLM with 470,000 individuals yielded correlations of 0.72, 0.57 and 0.30 for traits with 10,000 QTNs and heritabilities of 0.7, 0.5 and 0.2, respectively. That is, between 86% and 68% of the theoretical maximum, which is the square root of the heritability. We compared our results against predictions obtained from SNP effects computed using the BOLT-LMM software^8^, which is able to estimate variance components with large sample sizes on single compute nodes by performing approximations. The analyses with BOLT-LMM required up to ∼14 days to analyse a sample with 470,000 individuals using 8 threads in a single compute node. Compared with DISSECT, there was a significant decrease in the prediction accuracy of BOLT-LMM as the sample size of the training set increased (Supplementary Fig. 5). BOLT-LMM was designed and developed in the context of GWAS testing, where each marker effect is estimated independently, which could explain the loss of prediction accuracy that we observed.

We investigated why—even when training the models with this extremely large sample sizes—the limit of prediction accuracy was still not close to the theoretical maximum. As the estimation of QTN effects appeared to be very accurate (Supplementary Fig. 6), we hypothesized that the loss in accuracy might be a consequence of the improper QTN tagging by markers in the array. Under this hypothesis we expected that an array that included all the QTNs would substantially improve prediction accuracy.

### Prediction accuracy when all QTNs are genotyped

**Figure 4:**
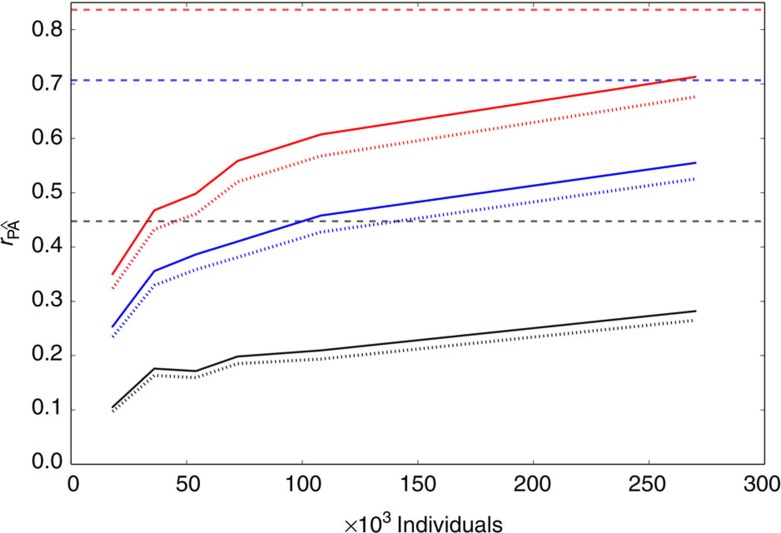
Prediction accuracy when all QTNs were genotyped. Correlation between true (P) and predicted phenotypes () as a function of the cohort size when the trait is determined by 10,000 QTNs. Black, blue and red curves represent traits with heritabilities of 0.2, 0.5 and 0.7, respectively. Solid lines are the correlations obtained when all QTNs were genotyped. Dotted lines are the correlations obtained when only ∼20% of QTNs were genotyped. Constant dashed lines indicate the maximum theoretical correlation for each heritability.

To test whether poor tagging of the QTNs explained the loss of accuracy, we assumed that all previously used tagging SNPs and all simulated QTNs were included in the genotyping array. Our results showed that the prediction accuracy for traits with 10,000 QTNs increased only slightly (Fig. 4). For traits with 1,000 QTNs the results were very similar to those of 10,000 QTNs (Supplementary Fig. 7). This suggests that under our genetic model one might not approach the theoretical limit of prediction accuracy even when training the models with 470,000 individuals and the genotyping array or resequencing included all QTNs^27^. We then hypothesized that if we were able to discriminate causal from non-causal variants, then prediction accuracy would improve. Hence, we repeated our experiments but now assuming that only the QTNs were included in the genotyping array. Prediction accuracy increased significantly (Supplementary Fig. 8), indicating that when the QTNs are included in the array, the noise introduced by other SNPs significantly reduces the accuracy of prediction obtained when fitting only the QTNs, even for very large number of individuals.

Finally, we checked our predictions with previously estimated theoretical prediction accuracies^16^,^27^ (Supplementary Fig. 9). Comparison with theoretical results in absolute terms may be difficult, as the exact values could depend on the parameters used in the model, which are unknown. In addition, theoretical results usually make unrealistic assumptions, such as that QTNs affecting the trait are independent of each other, that the SNPs genotyped are in linkage equilibrium, that the effect sizes are estimated individually, or that all QTNs are genotyped. However, our results agree with the predicted linear behaviour of the inverse of the squared correlation between the true and estimated genetic values as a function of the inverse of the sample size^16^,^27^. There are small discrepancies with the regression intercept, which is expected to be one. This could be due to several factors such as the limited number of points for the regression or the assumptions made in the theoretical approach.

## Discussion

Current software for performing genomic analysis with huge sample sizes on single compute nodes relies on performing approximations or compromises in terms of the complexity of the model fitted to the data^3^,^5^,^8^,^9^. Avoiding these constraints is a pressing need because of the fast increase of large genotyped cohorts such as the UK Biobank^28^. With this in mind, we have presented DISSECT, a new tool to perform a wide range of genetic and genomic analyses that overcomes these limitations by using the joint power of large numbers of networked computer nodes working together to perform the analyses. This approach also enables to reduce the computational times of the analyses by increasing the number of compute nodes. In addition, DISSECT will allow testing approximations with large sample sizes, because until now these approximations could only be tested with reduced sample sizes where the utility of the approximation are uncertain.

We demonstrated the power of DISSECT by addressing the timely topic of complex trait phenotypic prediction, which is of central interest to disciplines such as medicine, quantitative genetics or animal breeding. Prediction in unrelated humans has been an elusive goal^12^,^15^ due to a combination of suboptimal statistical methodology, small training data sets and lack of computational tools. DISSECT allowed us to fit MLMs to near 500,000 individuals and around 600,000 SNPs reaching prediction accuracies of up to 80% of the theoretical maximum on simulated quantitative traits. The increase of prediction accuracy when sample size increases has been predicted in previous theoretical work^16^,^27^,^29^,^30^. We have also shown that if all the QTNs are genotyped the noise introduced by non-causal SNPs could have a strong impact on the accuracy of prediction when using genomic best linear predictors (Supplementary Methods). This behaviour is consistent with previous work, which showed that including GWAS hits at more lenient significant thresholds could improve prediction accuracy^29^,^30^.

Although this demonstration of DISSECT's power concerned the problem of phenotypic prediction in humans, the software can also be used in plant and animal breeding, and perform a wide range of commonly used analyses. The main limitations are the availability of computing nodes and that a distributed scheme does not scale perfectly (that is, doubling the number of nodes does not exactly divide the computing time by half). DISSECT is under active development and there are several new functionalities planned or in testing stage (for example, GWAS adjusted by a polygenic effect and simultaneous fitting multiple variance components).

## Methods

### Simulations

We used the HAPGEN 2 software^31^ to simulate half a million individuals—based on linkage disequilibrium patterns and allele frequencies of 2,543,887 SNPs available in the Hapmap 2 (release 22) CEU population^32^—from which we generated subsets of 20, 40, 60, 80, 120, 300 and 500 thousand individuals. From each subset of data, we used 90% of the individuals for training the models and the rest for validating the predictions, except for the subset including 500,000 individuals where we used 470,000 individuals for training and 30,000 for validation. We simulated traits that were determined by 1,000 and 10,000 randomly distributed QTNs, respectively. The QTNs were randomly distributed across the genome and their combined effects explained 20, 50 and 70% of the phenotypic variation. That is, we simulated heritabilities (*h*^2^) of 0.2, 0.5 and 0.7. The QTNs effects were the same for all data subsets. Six replicates were performed for each trait heritability and genetic architecture, except for the subset including 500,000 individuals. Each replica assumed different QTNs with different effects drawn at random. The phenotypes were simulated using DISSECT, which assumes an additive genetic model for the selected QTNs (see Supplementary Note 1).

### MLM and prediction

MLM analyses were performed using DISSECT. The software and its source code are freely available (http://www.dissect.ed.ac.uk). For our first set of analyses we excluded all SNPs not present on the Illumina Human OmniExpress BeadChip. That gave us a set of 590,004 SNPs which included only ~20% of the QTNs available within the simulated data set. Later, we investigated the effect of having the QTNs in the genotyping array and included the remaining ∼80% of QTNs to the genotyping array.

The model fitted was:


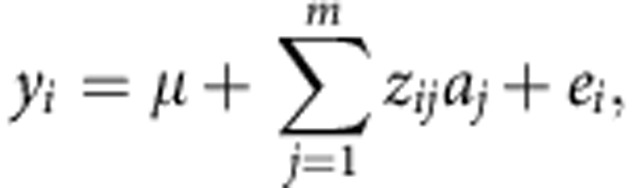


where *μ* is the mean term and *e*_*i*_ the residual. *z*_*ij*_ is the standardized genotype of individual *i* at marker *j*. The vector of random SNP effects **a** is distributed as 
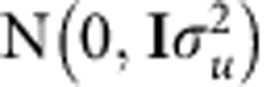
. 
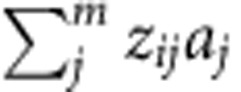
 is the total genetic effect for individual *i*. The phenotypic variance–covariance matrix is 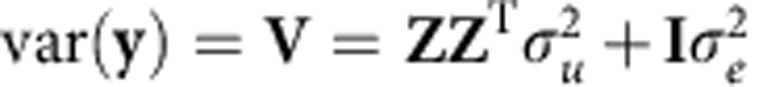. SNP effects were estimated using the equation^26^:


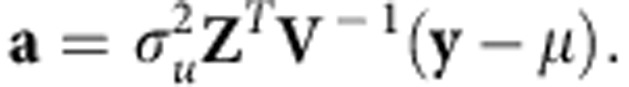


SNP effects were used as an input for DISSECT, to predict phenotypes on the validation cohort. DISSECT computes the prediction for individual *i* as a sum of the product of the SNP effects and the number of reference alleles of the corresponding SNPs:


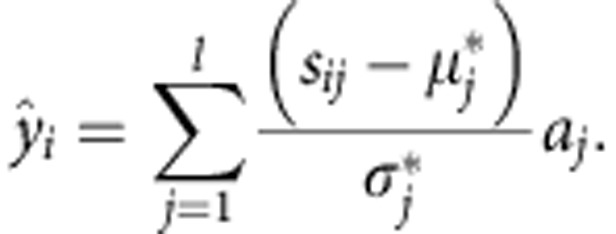


Where *s*_*ij*_ is the number of copies of the reference allele at SNP *j* of individual *i*, *l* is the number of SNPs used for the prediction and *a*_*j*_ is the effect of SNP *j* estimated from the MLM analyses or obtained from BOLT-LMM software^8^. 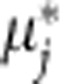 and 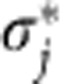 are the mean and the s.d. of the reference allele in the training population.

### Code availability

DISSECT source code is freely available under GPLv3 license at the url: http://www.dissect.ed.ac.uk.

## Additional information

**How to cite this article:** Canela-Xandri, O. *et al.* A new tool called DISSECT for analysing large genomic data sets using a Big Data approach. *Nat. Commun.* 6:10162 doi: 10.1038/ncomms10162 (2015).

## Supplementary Material

Supplementary Information Supplementary Figures 1-9, Supplementary Note 1 and Supplementary References
