# Supplementary material for: A new tool called DISSECT for analysing large genomic data sets using a Big Data approach
Source: Nat Commun. 2015 Dec 11;6:10162. doi: 10.1038/ncomms10162 (PMC4682108; doi:10.1038/ncomms10162)
Supplement: Supplementary Information — Supplementary Figures 1-9, Supplementary Note 1 and Supplementary References [file ncomms10162-s1.pdf]

Supplementary Figures

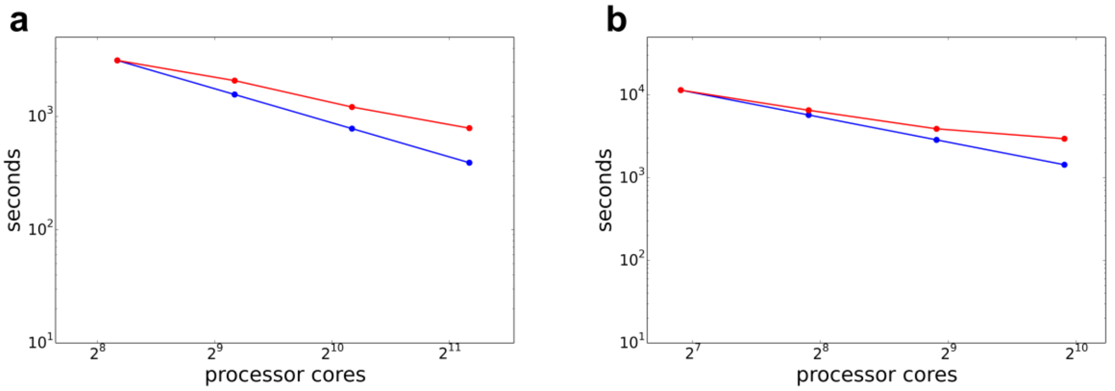

Supplementary Figure 1: **Scalability as a function of number of processor cores.** Computational time required for performing (**a**) Mixed Linear Model analysis with 108,000 individuals, and (**b**) principal component analysis with 72,000 individuals as a function of the number of processor cores used. The red line indicates the time used for the analysis whilst the blue line indicates the time predicted if scaling were perfect, i.e. if there were a perfect inverse relationship between processor core number and the time required to complete the analysis. We use as reference the left most point.

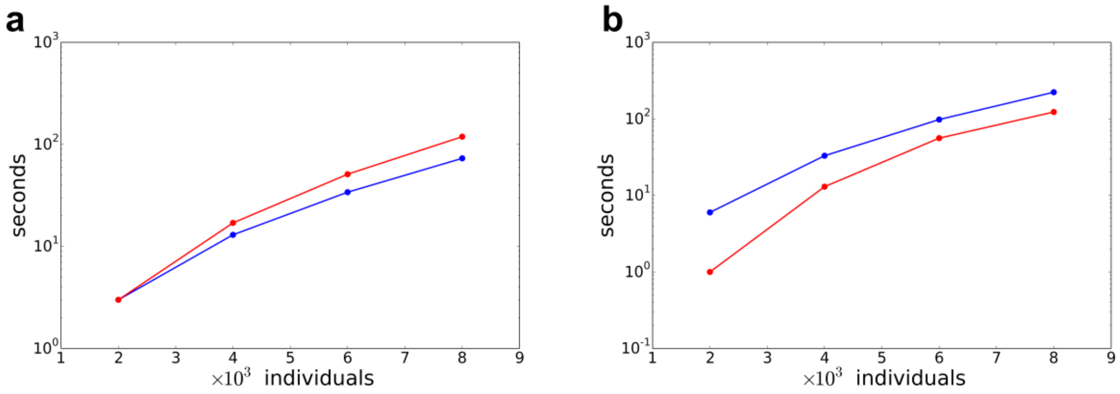

Supplementary Figure 2: **Computational performance in a single compute node.** The computational time required to perform the same (**a**) mixed linear model analysis and (**b**) principal component analysis as a function of sample size with DISSECT (blue line) and GCTA (red line). Computations were performed in a single compute node with small sample sizes.

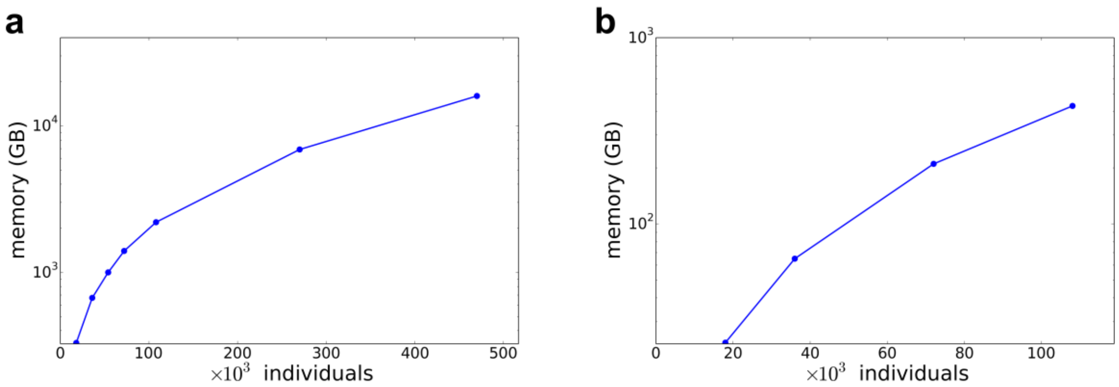

Supplementary Figure 3: **Memory usage as a function of sample size.**
Memory usage for (**a**) mixed linear model analysis and (**b**) principal component analysis as a function of sample size. Analysis parameters are identical to those used to generate Figure 2 in the main manuscript.

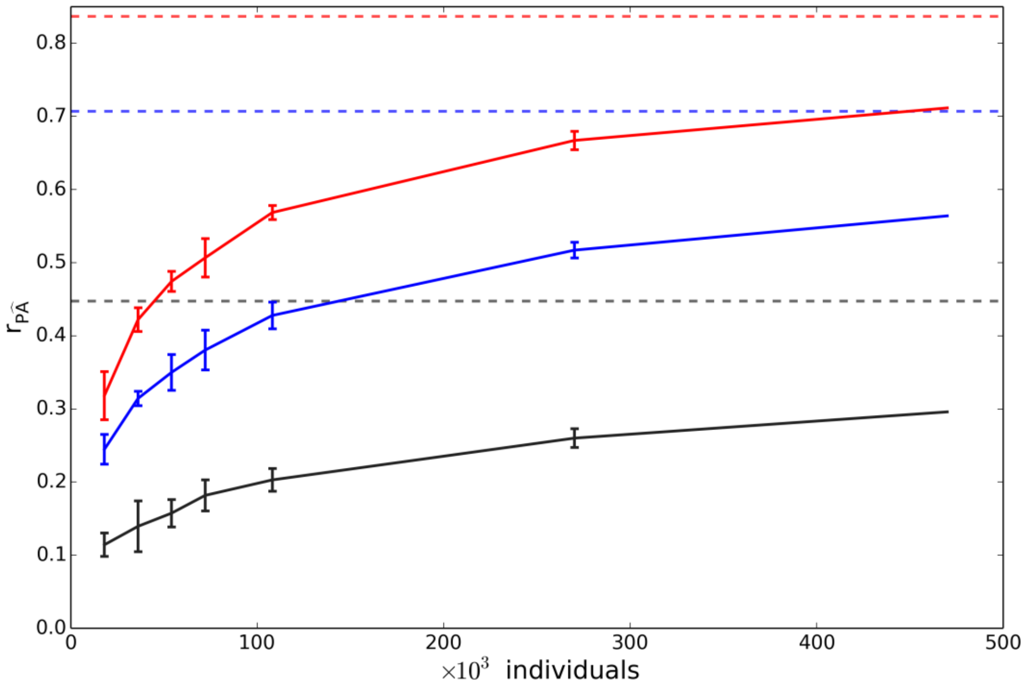

Supplementary Figure 4: **Prediction accuracy as a function of sample size.**
Correlation between true and predicted phenotypes as a function of cohort size for traits with 1,000 QTNs. Black, blue and red curves represent heritabilities of 0.2, 0.5, and 0.7, respectively. Constant dashed lines indicate the theoretical maximum achievable for each heritability. Error bars are two times the standard deviation over six replicas (470,000 individuals case has only one replica).

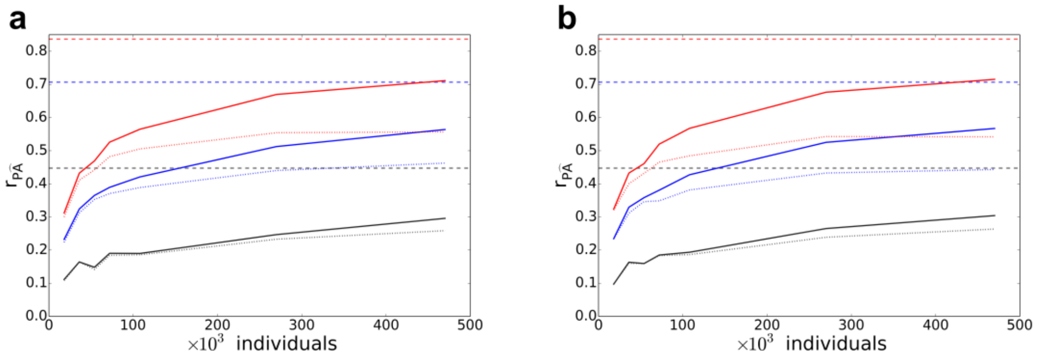

Supplementary Figure 5: **Prediction accuracy as a function of sample size. Comparison with BOLT-LMM.**

Correlation between true and predicted phenotypes as a function of cohort size for traits with (**a**) 1,000 QTNs and (**b**) 10,000 QTNs when only ~20% QTNs are genotyped. Black, blue and red curves represent heritabilities of 0.2, 0.5, and 0.7, respectively. Solid and dotted lines represent predictions using SNP effect sizes estimated using DISSECT and BOLT-LMM, respectively. Constant dashed lines indicate the theoretical maximum achievable for each heritability.

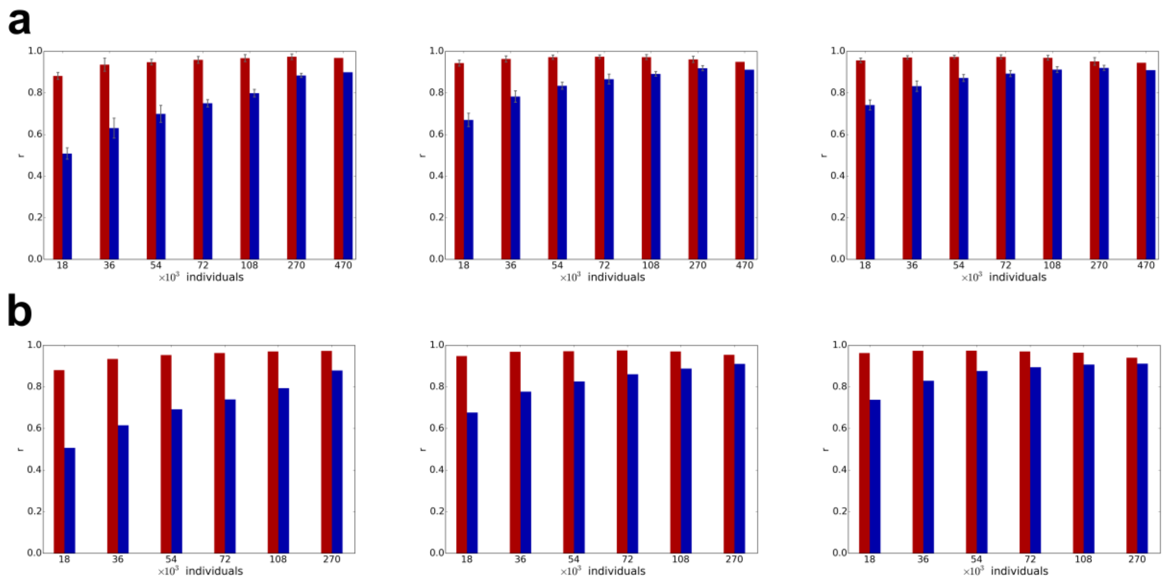

Supplementary Figure 6: Average correlation between estimated and true QTN effect sizes for traits with heritabilities of 0.2, 0.5, and 0.7 (from left to right).

Correlations when (**a**) only ~20% QTNs are genotyped, and (**b**) all QTNs are genotyped. For each cohort size, bars represent the correlation between true and estimated effects using the MLM analysis. Red and blue bars account for traits with 1,000 QTNs and 10,000 QTNs, respectively. Error bars are two times the standard deviation.

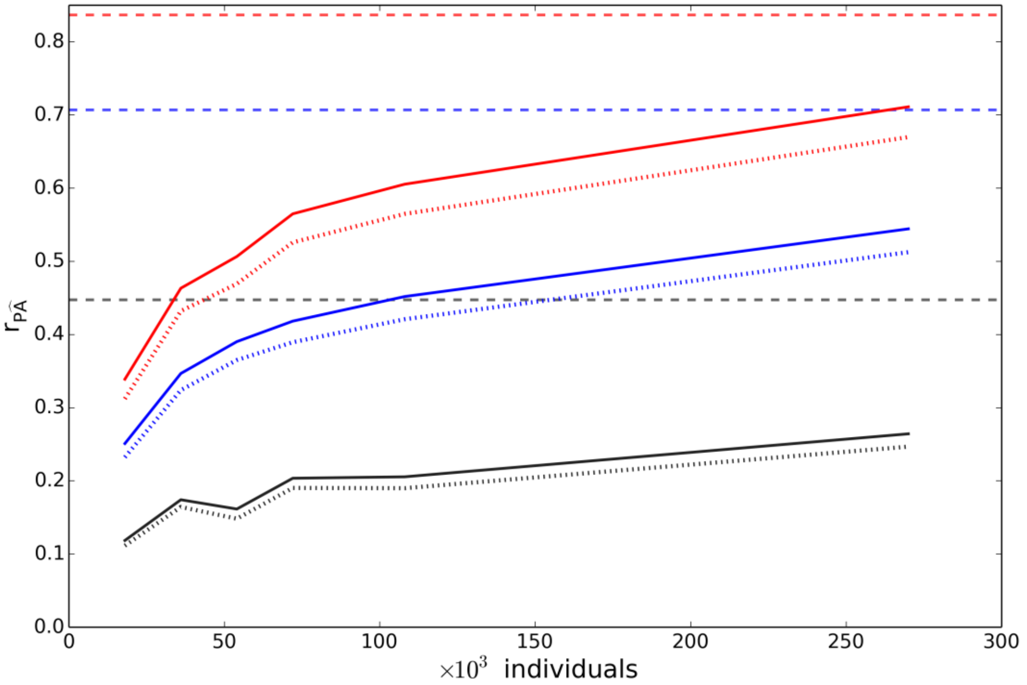

Supplementary Figure 7: Prediction accuracy when all QTNs are genotyped.
Correlation between true and predicted phenotypes as a function of cohort size for traits with 1,000 QTNs. Black, blue and red curves represent traits with heritabilities of 0.2, 0.5, and 0.7, respectively. Solid lines are the correlations obtained when all QTNs were genotyped. For comparison the results obtained when fitting the MLM to the tagging SNP array which contained ~20% of QTNs are shown with dotted lines. Constant dashed lines indicate the maximum theoretical correlation for each heritability.

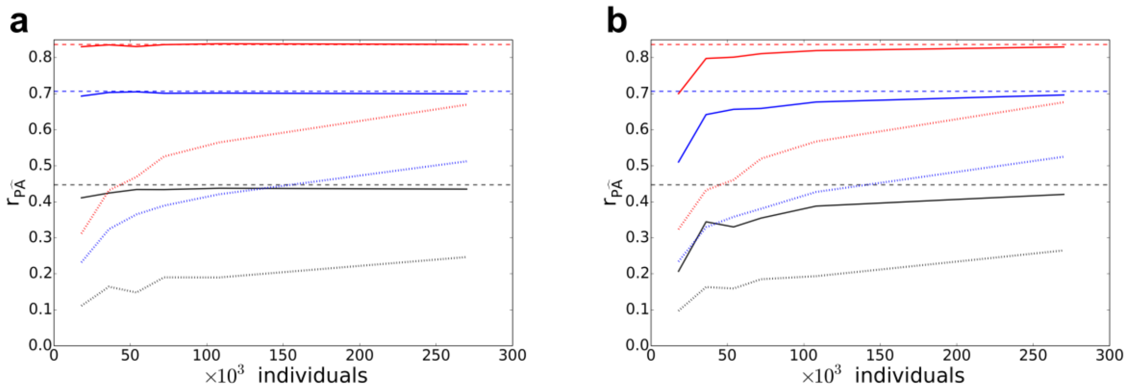

Supplementary Figure 8: Prediction accuracy when the genotyping array contains only the QTNs.
Accuracies for traits with (**a**) 1,000 QTNs and (**b**) 10,000 QTNs as a function of sample size. Solid lines represent the results of fitting the MLM to an array that only contained the QTNs. For comparison the results obtained when fitting the MLM to the tagging SNP array which contained ~20% of QTNs are shown with dotted lines. Black, blue and red colors represent traits with heritabilities of 0.2, 0.5, and 0.7, respectively. Constant dashed lines indicate the maximum theoretical correlation for each heritability.

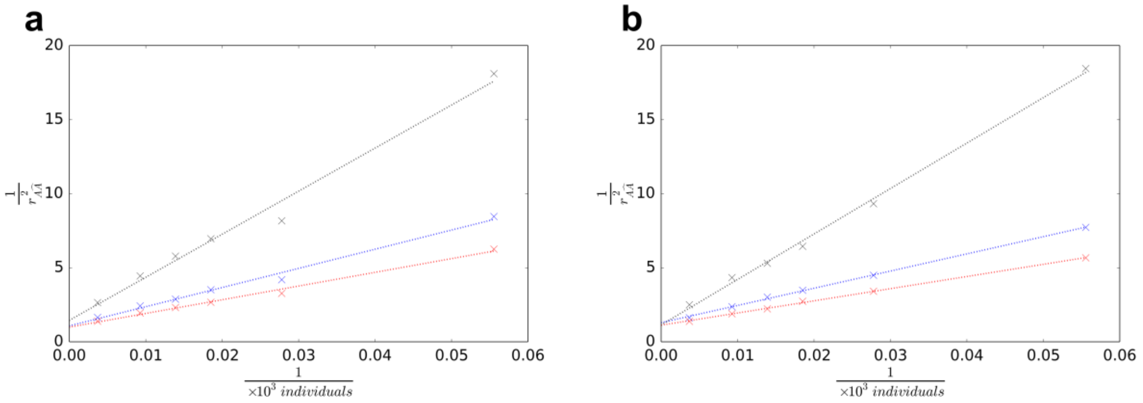

Supplementary Figure 9: **Comparison of DISSECT predictions with theoretical predictions.**

The inverse of the square of the correlation between the true (i.e. simulated) genetic effect and the predicted genetic effect as a function of the inverse of number of individuals for traits with (**a**) 1,000 QTNs and (**b**) 10,000 QTNs. Black, blue and red colors represent traits with heritabilities of 0.2, 0.5, and 0.7, respectively. Dashed lines display the fit of the points to a linear regression as expected by theoretical predictions (see main manuscript).

Supplementary Note 1

Using DISSECT

DISSECT is designed to be as easy to use as other commonly used genetic analysis software such as GCTA[1] or PLINK[2]. We provide precompiled versions for running on single compute nodes. On single compute nodes, DISSECT can be used by running the **dissect** command, supplying any required file paths or options as arguments or flags. A simple example of DISSECT use for computing a Genetic Relationship Matrix (GRM) would be:

dissect --bfile *genotypes* --make-grm --out *result*

However, DISSECT reaches its maximum potential on clusters of networked compute nodes. DISSECT will compile and run on any computational cluster with a correctly installed MPI implementation. The cluster size can range from a few compute nodes to hundreds or thousands of nodes, e.g. a supercomputer. Once DISSECT is installed, an analysis can be launched by running the same command as would be launched on a single node machine but prepending an MPI "runner" appropriate for the system on which the software is installed (**mpirun**, **aprun** or similar). DISSECT will automatically scale out to use the resources available to it. For example, the command to run the same GRM analysis as described above but on a multi-node cluster using 8 cores could be:

mpirun -n 8 dissect --bfile *genotypes* --make-grm --out *result*

The precise nature of (and flags for) the MPI runner depends on the specific cluster and MPI implementation (e.g. it could be "**mpirun --np 8**", "**aprun --np 8**", etc). In addition, many clusters require a submission script to be run to place the analyses into a queue system, where they must wait until there are sufficient computing resources available to run them.

A full list of the currently available options and input and output formats are published at the web site: http://www.dissect.ed.ac.uk. New updates and options will be published there.

The two-dimensional block-cyclic distribution

DISSECT performs distributed matrix computations using an efficient matrix distribution scheme known as two-dimensional block-cyclic distribution[3]. Under this scheme, a matrix is distributed as follows. First, a Block Size (BS) is defined (e.g. in the current version of DISSECT the default block size is $BS = 256$). Then, the matrix is divided in blocks of BS rows and BS columns, generating a matrix of blocks. We define $b_{r,c}$ as the block on row r and column c of the blocks matrix. Similarly, all the available compute processes (e.g. compute cores) have assigned a unique identification (ID) in a two dimensional grid, $p_{i,j}$. i being the row position of the process and j the column position. We define P_r and P_c as the number of processes in rows and in columns, respectively, i.e. the total number of processes is $P_r x P_c$. Then, the blocks in the first row of blocks ($b_{1,\cdot}$) are assigned to the first row of the grid of processes $p_{1,\cdot}$ cyclically in the following way. First column block, $b_{1,1}$, to first process column, $p_{1,1}$, second column block, $b_{1,2}$, to second process column, $p_{1,2}$, and so on until reaching the last process, P_c, in the current grid row. Then, we start again with the first processor column and repeat: block b_{1,P_c+1} is placed on process $p_{1,1}$, block b_{1,P_c+2} is placed on process $p_{1,2}$ and so one until reaching again the last process in the current grid row. We repeat this process until all the blocks in the first row of the blocks matrix are distributed in the first row of the grid of processes. The other rows of the blocks matrix are distributed following the same cyclic schema. The second row of blocks, $b_{2,\cdot}$ to second row of processes $p_{2,\cdot}$ the third row of blocks, $b_{3,\cdot}$ to third row of processes $p_{3,\cdot}$ and so on until we reach the last row of processes, P_r. Then, as in the columns, it starts again placing the $P_r + 1$ row of blocks matrix to the first row of processes, $p_{1,\cdot}$. Figure 1 of the main manuscript illustrates this distribution.

Distributing matrices following this schema compared to other more direct ways have some advantages. The most important one is that it usually allows a good load balancing by splitting the work reasonably evenly among the processes. For instance, with this distribution

when an algorithm needs to work in successive subparts of the matrix all processes still have a portion of a matrix of similar size to work on. This avoids having idle processors just waiting for others to finish, thus improving resource utilization and analysis speed-ups.

Algorithmic outline of a PCA analysis

DISSECT performs a wide range of analyses using distributed memory systems. To illustrate the basic working principles underlying the computational approach followed by DISSECT, we outline the steps performed for an exemplar computationally demanding analysis: Principal Component Analysis (PCA). For simplicity, in this description we assume very small data sizes, but the procedures remain representative of the more realistic large data sizes found in typical usage. Let's assume we have a genotype file with $M = 5$ SNPs, snp_i (i ranging from 1 to 5), and $N = 6$ individuals, ind_j (j ranging from 1 to 6). A major complexity associated with parallel programming for large distributed memory systems is that processes running on one node cannot directly access the memory of other processes running on other nodes, so the software is responsible to exchange the messages required for the multiple processes to cooperate on a single task. We here, again for simplicity, assume that we are performing the computation using only $P = 4$ processes, where each is resident on a different compute node. We assume that we are using a distribution with a block size of $BS = 2$ (see previous subsection). The main steps for a PCA analysis are:

1) DISSECT first initializes the communication between processes. Then, it sorts all the available processes into a two dimensional square grid (or, when P is not a square number, the code finds the two integer factors of P that straddle $\sqrt{P}$, e.g. an analysis with 24 processes will create a grid of 6x4 processes). In this simplistic description we will have a grid of 2x2 processes, were $p_{r,c}$ is the process in the row r and column c. The process $p_{1,1}$ is always set as the root process. Once the process

structure and communication has been setup, DISSECT parses all the command line options and starts the selected analysis, in our case, a PCA.

2) The root node loads the genotype metadata from the “.fam” and “.bim” files (i.e. the SNP names, positions, individual ids, etc) and stores this information in memory local to that node. The information about the total number of markers, M, and number of individuals, N, is broadcast to all the other processes. The genotype data in the “.bed” file can be interpreted as a matrix with the SNPs in the rows and the individuals in the columns. This data cannot be loaded on the root node and then distributed among the other processes because, for typical analyses, the corresponding data size is too large for the limited memory available on a single node (and furthermore, even without this limitation, it would be very slow to load into memory in such a serial fashion). To parallelize the genotype data loading process without saturating the hard disk access, the data is loaded by only the first column of the processes grid ($p_{1,1}$ and $p_{2,1}$ in our example) as follows. The matrix is divided on row blocks, rb_k, of size $BSxN$. That is, each row block, rb_k, includes a subset of BS SNPs for all the individuals. The process $p_{1,1}$ loads the first row block, rb_1, and the process $p_{2,1}$ the second, rb_2. Then, these processes compute the allele frequencies of the loaded SNPs and store them locally in their own memory. These processes also calculate the mapping of each genotype data element to its final location in the process grid. After this, they distribute the genotype data to the other processes of their same row by storing the data following a 2-dimensional block cyclic distribution. In this example, the process $p_{1,1}$ sends the corresponding blocks to the processes $p_{1,\cdot}$, and the process $p_{2,1}$ to the processes $p_{2,\cdot}$. When they have finished, they repeat for the following next two row blocks. That is, the process $p_{1,1}$ would read the third row block of SNPs, rb_3 from the genotypes file and the process $p_{2,1}$ the fourth row block, rb_4. Then, they would distribute the data again following the same method. This procedure is repeated until all the genotype data is loaded into a block-cyclic

distributed matrix, $\mathbf{G}$. After loading the genotype data, the processes in the first row $p_{1,\cdot}$, will contain the data for the SNPs, snp_1, snp_2, and snp_5 and the processes in the second row, $p_{2,\cdot}$ will contain the data for the SNPs, snp_3, and snp_4. The data for the individuals ind_1, ind_2, ind_5, ind_6 will be stored in the first column of processes, $p_{\cdot,1}$, and the data for the individuals 3 and 4 will be stored in the second column of processes, $p_{\cdot,2}$. The allele frequencies computed locally on the first column of processes while loading the data, are gathered and re-sorted into the root process, $p_{1,1}$. During that procedure, another matrix, $\mathbf{Q}$, is also created. It has the same dimensions and distribution as the genotype matrix, but contains a 0 for the genotypes that are missing and a 1 for the others.

3) After loading and distributing the data, the genotypes are standardised using the allele frequencies stored in the root node. To this end, the allele frequencies are distributed to the other processes according to the two dimensional cyclic distribution. That is, the allele frequencies for SNPs snp_1, snp_2, and snp_5 are sent to all the processes in the first processes row, $p_{1,\cdot}$, and the frequencies for SNPs snp_3, and snp_4 to all the processes in the second row, $p_{2,\cdot}$. Then, these frequencies are used by each process in local computations to subtract and divide on their local parts of the genotypes matrix the corresponding means and standard deviations computed from the allele frequencies. This operation transforms the matrix of genotypes $\mathbf{G}$ to a matrix with standardised genotypes $\mathbf{G}_S$.

4) After standardising the genotypes, the GRM can be computed. To this end, DISSECT first computes these two matrix products: $\mathbf{T}_1 = \mathbf{G}_S^T \cdot \mathbf{G}_S$ and $\mathbf{T}_2 = \mathbf{Q}_S^T \cdot \mathbf{Q}_S$. The component $t_{2_{ij}}$ of matrix $\mathbf{T}_2$ is the number of non-missing markers shared between the individuals i and j. To perform basic linear algebra operations like a basic matrix multiplication, DISSECT uses the standard ScaLAPACK libraries[3]. These are highly tested and accurate extensions of LAPACK libraries targeted to be used for software designed to run on distributed memory systems.

ScaLAPACK/LAPACK are used widely by many scientific software packages since this avoids unnecessary code redevelopment, ensures optimal performance for basic algebraic operations, and helps to prevent bugs. There are several implementations of these libraries and the specific algorithms utilized internally are implementation-dependent. However, they usually rely on recursive redistribution of matrix blocks of size $BSxBS$ between the different processes and calculation of the products block by block[4]. For instance, the resultant matrix will be also distributed in a block cyclic way. Then, if a block of the resultant matrix has to be stored in the process $p_{r,c}$, then this process collects all the required matrix blocks of the original matrices from the other processes and computes the resultant block. The cyclic block distribution is a scheme that permits an even distribution of the data between the processes. Once $\mathbf{T}_1$ and $\mathbf{T}_2$ are computed, the final GRM is computed by performing an element-wise division between $\mathbf{T}_1$ and $\mathbf{T}_2$. If both matrices have the same dimensions and are distributed using the same BS, as is the case, then this operation can be done by performing and element-wise division between the local elements of each matrix on each process.

5) Once the GRM is computed, then it may be filtered for pairs of individuals with a relatedness higher than a particular threshold or without at least a minimum of overlapping non-missing SNPs (where the thresholds depend on the used options). Since the matrices are distributed in a block-cyclic way, just deleting a single row or column requires a complete redistribution of all the data. So, this is an involved process that can require large communication resources as the data sizes become large. To optimize the filtering, first each process searches, on its local piece of the GRM, the elements that fulfill the requirements. Then, the local indices of these elements are translated to the global matrix indices and are broadcast to all the other processes. Therefore each process has an identical copy of all the global indices to be kept. Then, each process has to recompute the mappings of data elements to locations in the process grid for the new distribution, i.e where they have to send

each piece of their local matrix. They then repack all the local matrix elements that have to be sent to the other processes. After this, a global communication procedure takes place where all the processes send and receive these packs. Once each process receives its packs, they re-sort the data to end up with a block-cyclic copy of the filtered matrix.

6) The eigen decomposition of the filtered GRM is also a standard algebraic routine which is efficiently implemented by a ScaLAPACK function. The particular algorithmic details are implementation-dependent, usually rely on a matrix decomposition that works on smaller and smaller parts of the matrix like a Gaussian elimination process[3,5]. The cyclic-distribution scheme permits that when the algorithm advances and works on smaller matrix parts, all the processes have a relatively similarly-sized portion of the remaining matrix. As in the multiplication case, during this process, the operations are performed on the matrix blocks, and processes communicate to exchange blocks where necessary. At the end of the process, one ends up with a distributed matrix containing the eigen vectors and a diagonal matrix with the eigen values. In DISSECT, the diagonal matrices are special matrices which are stored as a standard single array in the root process.
7) Finally, the results are stored in the hard disk. To this end, the data of the eigen vectors that have to be stored are gathered from the distributed matrix into the root process. Then the root process combines the metadata of the individuals it already has with the received data to store the eigen vectors, and the eigen values (which are also in the root process) in a file.

The communication between processes is performed using the communication functions available from the MPI and/or BLACS libraries, depending on the particular case. These functions can send data from one process to another in different ways.

For simplicity in our above description, we only outlined the principal steps. The full implementation deals with a number of other factors and options such as filtering genotype

data, loading genotypes from different files and then joining them, computing the GRM by adding different GRMs computed from different genotype files, etc. More complex analyses such as fitting a MLM, require a much more intricate combination of operations and algorithms and more complex data structures. The steps performed are very dependent on the options used, but are computed using the basic principles described above: computations by matrix blocks of size $BSxBS$, communication between processes to share blocks, gathering or broadcasting data from the root process to the others, and use of the root process as the main output/input for small results or metadata, etc. When possible, the standard linear algebra computations are implemented through the ScaLAPACK library (e.g. standard matrix multiplications, inversions or eigen decompositions), which ensures optimal performance if using an optimized implementation. For other algebraic operations where the use of ScaLAPACK is not possible, or where specific optimizations are possible, we developed custom implementations (e.g. when performing element-wise operations, when computing the trace of a matrix product, when performing inversions, multiplications, or sums with at least one diagonal matrix involved, or when dealing with matrices made of diagonal blocks).

Phenotype Simulation

DISSECT performs phenotype simulations assuming an additive genetic model:

$$y_i = g_i + e_i = \sum_{j=1}^{M} z_{ij} u_j + e_i,$$

with y_i being the quantitative trait of individual i, u_j the effect of QTN j drawn from a normal distribution with zero mean and unit variance, M the number of assumed QTNs and e_i a normal distributed random variable with zero mean and variance $\sigma_g^2(1-\mathrm{h}^2)/\mathrm{h}^2$ where σ_g^2 is the variance of the genetic effect, g_i. z_{ij} is the standardised genotype of individual i at QTN j. It is defined as $z_{ij} = (s_{ij} - \mu_j)/\sigma_j$ where s_{ij} is the number of reference alleles at QTN j of

individual i, $\mu_j = 2p_j$ and $\sigma_j = \sqrt{2p_j(1-p_j)}$. μ_j and σ_j are the mean and the standard deviation of the marker j reference allele among the individuals genotyped, defined as a function of the reference allele frequency (p_j).

This analysis is not highly computationally demanding. The time complexity and memory requirements order is $O(MN)$, N being the sample size.

Genetic Relationship Matrix

DISSECT computes the genetic relationship matrix ($\mathbf{A}$) as,

$$a_{ij} = \frac{1}{M}\sum_{k=1}^{M} z_{ik}z_{jk},$$

with a_{ij} being the genetic relationship between individuals i and j. M is the number of markers. z_{ik} is the standardised genotype of individual i at marker k as shown in the previous section.

DISSECT can also compute and store the eigen decomposition of the genetic relationship matrix. This matrix can be used as an input for fitting Mixed Linear Models much faster (e.g. when only estimating the genetic variance and the environmental variance).

This analysis becomes highly computationally demanding when the number of individuals, N, increases. The asymptotic time complexity is $O(MN^2)$ and the memory requirements depends on the method used for loading the genotypes and computing the genetic relationship matrix. The memory requirements are of the order of $O(MN)$ for loading genotype data and $O(N^2)$ for the storing the genetic relationship matrix. When $M \gg N$, the majority of the memory is used for storing the genotypes. In this situation, memory requirements can be reduced considerably by loading the genotypes by parts, computing the genetic relationship matrix for each part, and then adding all parts together[6] which have a usually negligible time complexity of $O(N^2)$. DISSECT uses this approach when the input

genotypes are a group of files containing different subsets of SNPs (i.e. SNPs grouped by chromosomes). The time complexity of performing the eigen decomposition of the genetic relationship matrix is $O(N^3)$.

Univariate Mixed Linear Models

DISSECT can fit mixed linear models using the model,

$$y_i = \mu + \sum_{l=1}^{L} x_{il}\beta_l + \sum_{j=1}^{M} z_{ij}a_j + e_i,$$

where μ is the mean term and e_i the residual for individual i. L is the number of fixed effects, x_{il} being the value for the fixed effect l at individual i and β_l the estimated effect of the fixed effect l. M is the number of markers and z_{ij} is the standardised genotype (see the Phenotype Simulation section) of individual i at marker j. The vector of random SNP effects $\mathbf{a}$ is distributed as $\mathrm{N}(0, \mathbf{I}\sigma_u^2)$. The phenotypic variance-covariance matrix is $\mathrm{var}(\mathbf{y}) = \mathbf{V} = \mathbf{Z}\mathbf{Z}^{\mathrm{T}}\sigma_u^2 + \mathbf{I}\sigma_e^2$. It estimates SNP effects using the equation[7]:

$$\mathbf{a} = \sigma_u^2\mathbf{Z}^T\mathbf{V}^{-1}(\mathbf{y} - \boldsymbol{\mu} - \mathbf{X}\boldsymbol{\beta}).$$

Because $\sum_j^m z_{ij}a_j$ is the total additive genetic effect (g_i) for individual i, this model can also be expressed as,

$$y_i = \mu + \sum_{l=1}^{L} x_{il}\beta_l + g_i + e_i.$$

In this model, the vector of genetic effects $\mathbf{g}$ is distributed as $\mathrm{N}(0, \mathbf{A}\sigma_g^2)$. Where $\mathbf{A}$ is the genetic relationship matrix and $\sigma_g^2 = M\sigma_u^2$. Accordingly, the total phenotypic variance-covariance matrix is $\mathrm{var}(\mathbf{y}) = \mathbf{V} = \mathbf{A}\sigma_g^2 + \mathbf{I}\sigma_e^2$. From the equivalence between these two models, DISSECT can estimate the total additive effect from the equation:

$$\mathbf{g} = \sigma_g^2\mathbf{A}\mathbf{V}^{-1}(\mathbf{y} - \boldsymbol{\mu} - \mathbf{X}\boldsymbol{\beta}).$$

DISSECT fits σ_g^2 and σ_e^2 using the expectation maximization (EM) method for the first step[7], followed by AI REML method steps[8,9]. For this analysis, diagonal genetic relationship

matrices can be used. This greatly reduces the computational time required for performing the analysis.

This analysis becomes highly computationally demanding when the number of individuals, N, increases. The asymptotic time complexity and memory requirements depend on the options used for the analysis. If the genetic relationship matrix is precomputed in a previous analysis the time complexity is $O(N^3)$ and memory requirements are of the order of $O(N^2)$. In case where the genetic relationship matrix has to be computed prior to starting the analysis, then the memory and computational requirements increase as explained in the previous section. In addition, some options could have a significant impact on computational and memory requirements. In particular, if the genetic relationship matrix has already been computed, but DISSECT has to estimate the SNP effects, then genotype data have to be loaded. In this situation, the memory requirements can increase considerably. The requirements for loading the genotype data are of the order of $O(MN)$.

The time complexity of fitting the mixed linear model can be reduced to $O(N)$ when the analysis is performed using a diagonalized genomic relationship matrix. Diagonalizing the matrix has a computational complexity similar to that of fitting the mixed linear model without a diagonalized matrix. However, when several analyses are to be performed with the same genetic relationship matrix (e.g. when using different traits or fixed effects), then the benefits of using a pre-calculated diagonalized genetic relationship matrix can be considerable.

Bivariate Mixed Linear Models

DISSECT can fit bivariate mixed linear models defined by the equation[10],

$$\mathbf{y} = \begin{pmatrix}\mathbf{y}_1\\ \mathbf{y}_2\end{pmatrix} = \begin{pmatrix}\boldsymbol{\mu}_1\\ \boldsymbol{\mu}_2\end{pmatrix} + \begin{pmatrix}\mathbf{X}_1 & 0\\ 0 & \mathbf{X}_2\end{pmatrix}\begin{pmatrix}\boldsymbol{\beta}_1\\ \boldsymbol{\beta}_2\end{pmatrix} + \begin{pmatrix}\mathbf{g}_1\\ \mathbf{g}_2\end{pmatrix} + \begin{pmatrix}\mathbf{e}_1\\ \mathbf{e}_2\end{pmatrix},$$

where $\boldsymbol{\mu}_i$ is a vector of equal mean terms and $\mathbf{e}_i$ the vector of residuals for the trait i. $\mathbf{X}_i$ is the incidence matrix of the fixed effects $\boldsymbol{\beta}_i$ for the trait i. $\mathbf{g}_i$ is the vector of individuals genetic effects for the trait i with covariance matrix:

$$\mathrm{var}\begin{pmatrix}\mathbf{g}_1\\ \mathbf{g}_2\end{pmatrix} = \begin{pmatrix}\mathbf{A}_1\sigma_{g_1}^2 & \mathbf{A}_{12}\sigma_{g_1g_2}\\ \mathbf{A}_{12}^T\sigma_{g_1g_2} & \mathbf{A}_2\sigma_{g_2}^2\end{pmatrix},$$

where $\mathbf{A}_i$ is the genetic relationship matrix between the individuals measured for trait i and $\mathbf{A}_{ij}$ the genetic relationship matrix between the individuals measured for trait i and trait j. $\sigma_{g_1}^2$, $\sigma_{g_2}^2$, and $\sigma_{g_1g_2}$ are the genetic variance for trait 1, the genetic variance for trait 2 and the genetic covariance between both traits, respectively. The total covariance matrix ($\mathbf{V}$) reads,

$$\mathrm{var}(\mathbf{y}) = \mathbf{V} = \begin{pmatrix}\mathbf{A}_1\sigma_{g_1}^2 & \mathbf{A}_{12}\sigma_{g_1g_2}\\ \mathbf{A}_{12}^\mathrm{T}\sigma_{g_1g_2} & \mathbf{A}_2\sigma_{g_2}^2\end{pmatrix} + \begin{pmatrix}\mathbf{I}\sigma_{e_1}^2 & \mathbf{I}_{12}\sigma_{e_1e_2}\\ \mathbf{I}_{12}^T\sigma_{e_1e_2} & \mathbf{I}\sigma_{e_2}^2\end{pmatrix},$$

where $\sigma_{e_1}^2$, $\sigma_{e_2}^2$, and $\sigma_{e_1e_2}$ are the environmental variance for trait 1, the environmental variance for trait 2 and the environmental covariance between both traits, respectively. $\mathbf{I}$ is the identity matrix and $\mathbf{I}_{12}$ is a matrix where the elements in row i and column j are 1 if the individual i for the trait 1 is the same than the individual j of the trait 2 and 0 otherwise. DISSECT fits the variances and covariances using the expectation maximization (EM) method for the first step[7], followed by AI REML method steps[8,9]. For this analysis, when the individuals for both traits are the same, diagonal genetic relationship matrices can be used to significantly reduce the computational time required for the analysis.

Memory and time complexity are the same as for the univariate mixed linear models assuming a sample size which is equal to the total sum of all individuals in each trait.

Regional Mixed Linear Models

DISSECT can use regional mixed linear models for studying the accumulated variance explained by the alleles within genomic regions[6,11]. In this model, SNPs are divided in K different regions. For each region (k) the SNPs can be divided between those inside the region, R_k, and those outside the region, G_k. All regions are fitted recursively using the model:

$$y_i = \mu + \sum_{l=1}^{L} x_{il}\beta_l + g_i^{R_k} + g_i^{G_k} + e_i,$$

where μ is the mean term and e_i the residual for individual i. L is the number of fixed effects, x_{il} is the value for the fixed effect l at individual i, and β_l the estimated effect of fixed effect l. $g_i^{R_k}$ and $g_i^{G_k}$ are the total genetic effect in region k attributable to SNPs in groups R_k and G_k, respectively. The vectors of genetic effects $\mathbf{g}^{R_k}$ and $\mathbf{g}^{G_k}$ are distributed as $\mathrm{N}\left(0, \mathbf{A}^{R_k}\sigma^2_{g^{R_k}}\right)$ and $\mathrm{N}\left(0, \mathbf{A}^{G_k}\sigma^2_{g^{G_k}}\right)$, respectively. $\mathbf{A}^{G_k}$ and $\mathbf{A}^{R_k}$ are the genetic relationship matrices computed using only the SNPs present in the corresponding group. The variances $\sigma^2_{g^{R_k}}$ and $\sigma^2_{g^{G_k}}$ are the estimated genetic variance explained by the SNPs on these groups. Under this model, the phenotypic variance-covariance matrix is $\mathrm{var}(\mathbf{y}) = \mathbf{V} = \mathbf{A}^{R_k}\sigma^2_{g^{R_k}} + \mathbf{A}^{G_k}\sigma^2_{g^{G_k}} + \mathbf{I}\sigma^2_e$. DISSECT fits the variances and covariances using the expectation maximization (EM) method for the first step[7], followed by AI REML method steps[8,9].

Memory and time complexity is similar to those for the univariate mixed linear model case. The main differences are that the model must be fitted for each region, that genotype data must be loaded (memory requirements are of the order of $O(MN)$), and that for each region, genetic relationship matrices must be subtracted (which have a negligible time complexity of $O(N^2)$).

AI REML Method

The AI REML method[8,9] is an iterative method for estimating the variances on a mixed linear model. It is based in the following iterative equation,

$$\mathbf{\Theta}^{(k+1)} = \mathbf{\Theta}^{(k)} + \mathbf{AI}^{-1}\left.\frac{\partial L}{\partial \mathbf{\Theta}}\right|_{\mathbf{\Theta}^{(k)}},$$

where $\mathbf{\Theta}^{(k)}$ is the vector of the estimated variance components at step k. $\partial L/\partial \mathbf{\Theta}$ is a vector of the partials of the log-likelihood function with respect the components in $\mathbf{\Theta}$. It can be computed as,

$$\frac{\partial L}{\partial \sigma_i^2} = -\frac{1}{2}\mathrm{tr}\left(\mathbf{P}\frac{\partial \mathbf{V}}{\partial \sigma_i^2}\right) + \frac{1}{2}\left(\mathbf{y}^T\mathbf{P}\frac{\partial \mathbf{V}}{\partial \sigma_i^2}\mathbf{P}\mathbf{y}\right),$$

$\mathbf{V}$ being the covariance matrix, $\mathbf{y}$ the vector of phenotypes and $\mathbf{P}$ is defined as,

$$\mathbf{P} = \mathbf{V}^{-1} + \mathbf{V}^{-1}\mathbf{X}\left(\mathbf{X}^{T}\mathbf{V}^{-1}\mathbf{X}\right)^{-1}\mathbf{X}^{T}\mathbf{V}^{-1},$$

where $\mathbf{X}$ is the incidence matrix of the fixed effects. The $\mathbf{AI}$ matrix is the average information matrix and it is defined as,

$$\mathbf{AI}_{ij} = \frac{1}{2}\left[\mathbf{y}^{T}\mathbf{P}\frac{\partial\mathbf{V}}{\partial\sigma_i^2}\mathbf{P}\frac{\partial\mathbf{V}}{\partial\sigma_j^2}\mathbf{Py}\right].$$

Expectation Maximization Method

The Expectation Maximization method[7] is an iterative method for estimating the variances on a mixed linear model. It is based in the following iterative equation,

$$\left(\sigma_i^2\right)^{(k+1)} = \left(\sigma_i^2\right)^{(k)} + \frac{1}{N}\left(\sigma_i^4\right)^{(k)}\left[\left(\mathbf{y}^{T}\mathbf{P}\frac{\partial\mathbf{V}}{\partial\sigma_i^2}\mathbf{Py}\right) - \mathrm{tr}\left(\mathbf{P}\frac{\partial\mathbf{V}}{\partial\sigma_i^2}\right)\right],$$

where $\left(\sigma_i^2\right)^{(k)}$ is the the estimated variance at step k, N the sample size, $\mathbf{V}$ the covariance matrix and $\mathbf{P}$ is defined in the previous section.

Principal component analysis

In order to perform principal component analysis, DISSECT computes the genetic relationship matrix (see the Genetic Relationship Matrix section) and then performs an eigen decomposition of the matrix.

This analysis becomes highly computationally demanding when the number of individuals, N, increases. The asymptotic time complexity and memory requirements depends on the options used for the analysis. If the genetic relationship matrix is precomputed in a previous analysis the time complexity is $O(N^3)$ and memory requirements are of the order of $O(N^2)$. If the genetic relationship matrix needs to be computed before starting the analysis, then the memory and computational requirements are incremented as explained in the Genetic Relationship Matrix section.

Genome-wide association study

DISSECT carries out GWAS by regressing the standardised SNP genotypes on the quantitative trait using the following linear regression model for testing SNP j:

$$y_i = \mu + \sum_{l=1}^{L} x_{il}\beta_l + z_{ij}a_j + e_i,$$

where μ is the mean term, z_{ij} is the standardised genotype (see the Phenotype Simulation section) of individual i at SNP j, the slope a_j is the effect of SNP j, and e_i is the residual. L is the number of covariates, being x_{il} the value for the covariate l at individual i and β_l its estimated effect. DISSECT can also recursively fit models where SNPs are fitted in groups:

$$y_i = \mu + \sum_{l=1}^{L} x_{il}\beta_l + \sum_{j \in G_k} z_{ij}a_j + e_i,$$

where G_k is a set containing the indices of all SNPs in the group k. All groups are fitted recursively.

Prediction

SNP effects can be used as an input for DISSECT to predict phenotypes on an independent cohort. DISSECT computes the prediction for individual i as a sum of the product of the SNP effects and the number of reference alleles of the corresponding SNPs:

$$\hat{y}_i = \sum_{j=1}^{M} \frac{(s_{ij} - \mu_j^*)}{\sigma_j^*} a_j$$

Where s_{ij} is the number of copies of the reference allele at SNP j of individual i, M is the number of SNPs used for the prediction, and a_j the effect of SNP j. μ_j^* and σ_j^* are the mean and the standard deviation of the reference allele in the training population.

This analysis is not highly computationally demanding. The order of the time complexity and memory requirements is $O(MN)$, N being the sample size.

Supplementary References

1. Yang, J., Lee, S. H., Goddard, M. E. & Visscher, P. M. GCTA: a tool for genome-wide complex trait analysis. *Am. J. Hum. Genet.* **88,** 76–82 (2011).

2. Purcell, S. *et al.* PLINK: a tool set for whole-genome association and population-based linkage analyses. *Am. J. Hum. Genet.* **81,** 559–75 (2007).

3. Blackford, L. S. *et al. ScaLAPACK Users' Guide*. (Society for Industrial and Applied Mathematics, 1997).

4. Jaeyoung Choi. A new parallel matrix multiplication algorithm on distributed-memory concurrent computers. in *Proceedings High Performance Computing on the Information Superhighway. HPC Asia '97* 224–229 (IEEE Comput. Soc. Press, 1997). doi:10.1109/HPC.1997.592151

5. Choi, J., Dongarra, J. J. & Walker, D. W. The design of a parallel dense linear algebra software library: Reduction to Hessenberg, tridiagonal, and bidiagonal form. *Numer. Algorithms* **10,** 379–399 (1995).

6. Cebamanos, L., Gray, A., Stewart, I. & Tenesa, A. Regional heritability advanced complex trait analysis for GPU and traditional parallel architectures. *Bioinformatics* **30,** 1177–1179 (2014).

7. Lynch, M. & Walsh, B. *Genetics and Analysis of Quantitative Traits*. (Sinauer, 1998).

8. Gilmour, A. R., Thompson, R. & Cullis, B. R. Average Information REML: An Efficient Algorithm for Variance Parameter Estimation in Linear Mixed Models. *Biometrics* **51,** 1440 (1995).

9. Lee, S. H. & van der Werf, J. H. J. An efficient variance component approach implementing an average information REML suitable for combined LD and linkage mapping with a general complex pedigree. *Genet. Sel. Evol.* **38,** 25–43 (2006).

10. Lee, S. H., Yang, J., Goddard, M. E., Visscher, P. M. & Wray, N. R. Estimation of pleiotropy between complex diseases using single-nucleotide polymorphism-derived genomic relationships and restricted maximum likelihood. *Bioinformatics* **28,** 2540–2 (2012).

11. Nagamine, Y. *et al.* Localising loci underlying complex trait variation using Regional Genomic Relationship Mapping. *PLoS One* **7,** e46501 (2012).
